# Psychosocial Correlates of Sunburn among Young Adult Women

**DOI:** 10.3390/ijerph9062241

**Published:** 2012-06-18

**Authors:** Carolyn J. Heckman, Susan Darlow, Jessye Cohen-Filipic, Jacqueline D. Kloss, Teja Munshi, Clifford S. Perlis

**Affiliations:** 1 Cancer Prevention and Control Program, Fox Chase Cancer Center, 333 Cottman Avenue, Philadelphia, PA 19111, USA; Email: susan.darlow@fccc.edu (S.D.); teja.munshi@fccc.edu (T.M.); clifford.perlis@fccc.edu (C.S.P.); 2 Portland VA Medical Center, 3810 SW U.S. Veterans Hospital Road, Portland, OR 97239, USA; Email: jessyecohen@gmail.com; 3 Department of Psychology, Drexel University, 3141 Chestnut Street, Philadelphia, PA 19104, USA; Email: jdk29@drexel.edu; 4 Cancer Prevention and Control Program, The Cancer Institute of New Jersey, 195 Little Albany Street, New Brunswick, NJ 08901, USA; Email: mannesl@umdnj.edu

**Keywords:** sunburn, young adult women, psychosocial correlates, sunscreen, Health Beliefs Model, skin cancer prevention

## Abstract

Skin cancer is an increasingly common disease, particularly among young adult women. Sunburn early in life is a risk factor for skin cancer. Few studies have reported on psychosocial correlates of sunburn. The current study consisted of an online survey of undergraduate women from a university in the northeastern part of the USA. A logistic regression demonstrated that young women who reported a history of four or more sunburns were significantly more likely to report fair skin, higher perceived susceptibility to skin cancer, greater perceived benefits of tanning (e.g., appearance enhancement), lower perceived control over skin protection, and more frequent sunscreen use. Sunbathing was not associated with a greater number of sunburns. These results suggest that young women who sunburn more often possess other skin cancer risk factors, are aware of their susceptibility to skin cancer, and try to use sunscreen, but feel limited control over their skin protection behavior and are not less likely to sunbathe than others. Therefore, interventions are needed to assist high risk young women in asserting more control over their sun protection behavior and perhaps improve the effectiveness of the sunscreen or other skin protection methods they do employ.

## 1. Introduction

Skin cancer is the most common form of cancer in the USA, accounting for half of all human malignancies, with over two million new cases diagnosed yearly [1]. The incidence of skin cancer has been rising faster than that of any other cancer [2]. In recent years, the incidence of melanoma, the most deadly form of skin cancer, has more than doubled among young women aged 15–29 years, from 5.5 to 13.9 cases per 100,000 [3]. While most skin cancers are not fatal, they are becoming increasingly common, costly, and can have devastating effects on health and appearance. 

Sixty to ninety percent of all melanomas can be attributed to ultraviolet (UV) radiation [4,5], and UV is considered the single most significant modifiable risk factor in the prevention of melanoma [2]. Sunburns increase the risk of melanoma, particularly repeated blistering sunburns during childhood and adolescence [6,7]. Sunburn during childhood can double the risk for the development of melanoma in adulthood [8,9]. A recent review of national studies found that the incidence of past year sunburn among adolescents ages 11–18 years was 68.7% [10]. One in three US adults also reports burning during the past year, with rates being highest among young adults aged 18–29 years (57.5%) [10]. 

Studies of the correlates of sunburn have focused on demographic variables such as gender, race, and age, with most of these studies finding that young white women are most likely to report a history of sunburns [10,11,12,13]. Few studies have examined psychosocial correlates of sunburn. Studies of USA and European adolescents and adults found associations between more frequent sunburn and positive attitudes towards tanning and tanned skin, as well as having a social network who tans [14,15,16,17]. A national study of US adolescents found a positive association between sunburn and sunscreen use [18]. A recent study found that perceived susceptibility to burns was associated with burning during indoor tanning [19].

No study, to our knowledge, has applied a theoretical model to predicting sunburns. The Health Belief Model addresses motivational factors for behaviors associated with disease risk, such as perceived susceptibility to and severity of diseases such as skin cancer, perceived benefits of and barriers to associated behaviors (e.g., tanning and skin protection), self-efficacy and control over behaviors, and behavioral intentions [20]. HBM variables have been used to predict sunbathing and sun protection behaviors but not sunburn *per se* [21,22,23]. Examining psychosocial variables associated with sunburn may be valuable given the prevalence of sunburns and the increased risk of melanoma in those who have experienced sunburn, particularly during childhood and early adulthood [6].

The purpose of the current study was to explore potential correlates of sunburn in a high risk population of young adult women. The variables included in the analyses were biological (fair skin, family history of skin cancer), attitudinal from the HBM (perceived benefits of tanning and barriers to sunscreen use, perceived severity of and susceptibility to skin cancer and damage, self-efficacy for skin protection, perceived control over tanning and skin protection, and intentions to avoid tanning and protect the skin), and behavioral (sunbathing and sunscreen use). 

## 2. Methods

### 2.1. Participants

Female undergraduates at a university (*N* = 509: 93% of the 546 individuals who initially consented for the study) in the northeastern part of the USA completed the questionnaires. Participants’ ages ranged from 18 to 25 years (*M* = 19.8, SD = 1.6). Racial distribution was as follows: 66.5% White, 16.2% Asian-American, 12.2% Other/Mixed, and 4.9% Black/African-American. Four percent of the sample identified as Hispanic or Latino.

### 2.2. Measures

#### 2.2.1. Biological Factors

Participants were asked to indicate the color of their untanned skin, with possible choices being very fair, fair, medium light, medium (olive), medium dark, and very dark. This is one item used to help determine Fitzpatrick skin type [24,25]. Participants who indicated that they had very fair or fair skin were categorized as fair-skinned (due to greater risk of skin cancer and sunburn in those with fair skin). All others were considered darker than fair-skinned. Participants were also asked if they had a family member with skin cancer.

#### 2.2.2. Perceived Severity and Susceptibility

Perceived health and emotional impact severity of skin cancer and photo-aging was assessed using a four-item measure by Aiken and colleagues [26]. Items were scored on a 1 to 5 Likert-type scale of agreement (e.g., “The health consequences of developing skin cancer are severe”). This measure demonstrated adequate internal consistency in our study (α = 0.67). Perceived susceptibility to skin cancer was assessed using one item in which participants were asked to indicate their perceived chances of getting skin cancer in the future, compared to other women their own age from 1 = much less to 5 = much more [27].

#### 2.2.3. Benefits of and Barriers to Tanning and Sun Protection

Participants completed three items designed to assess perceived benefits (e.g., for appearance) of tanning (α = 0.88). These items were from Gibbons and colleagues [28], and were scored on a five-point Likert-type scale of agreement (e.g., “Having a tan improves the way most people look”). Perceived barriers (e.g., forgetting) to using sunscreen was assessed using five items adapted from Mahler, Kulik, Gibbons, Gerrard and Harrell [29]. All items (e.g., “Sunscreen is more trouble than it’s worth”) were scored on a five-point Likert-type scale, which was internally consistent (α = 0.70) in our study sample.

#### 2.2.4. Self-Efficacy and Perceived Control over Tanning and Sun Protection

Self-efficacy for using various types of sun protection (e.g., sunscreen, protective clothing) was assessed using a seven-item measure with items scored on a five-point Likert-type scale of agreement [30]. Participants indicated how confident they were that they could engage in sun protection-related behaviors such as “Use sunscreen whenever you are out in the summer for more than 15 minutes”. This measure was internally consistent in our study (α = 0.81). For perceived controllability, participants were asked to indicate how easy or difficult it would be, on a 7 point scale (“1” being “Very difficult”, “7” being “Very easy”), to not intentionally sunbathe and to consistently protect their skin from UV radiation [31].

#### 2.2.5. Behavioral Intentions

Behavioral intentions to avoid tanning and protect the skin from UV radiation were assessed using nine items from Mahler and colleagues [32], all scored on a 7-point Likert-type scale. Intentions to protect the skin from UV radiation was assessed using seven items pertaining to different aspects of skin protection (e.g., sunscreen, protective clothing), with robust internal consistency in our study (α = 0.80). Intentions to avoid tanning were assessed using two items, specifically to “avoid indoor tanning” and “avoid intentionally sunbathing.”

#### 2.2.6. Behavioral Factors

Participants were asked to indicate how many times in their entire lives they have had a red or painful sunburn that lasted a day or more [25]. Using a median split, we categorized participants as having sunburned three or fewer times, or four or more times. This was our outcome variable.

Other behaviors assessed were sunbathing and sunscreen use. Participants indicated how many days on average they spend tanning in the sun each week [33]. Participants were dichotomized into those who spend at least one day a week tanning in the sun *versus* those who do so less than one day a week. Participants indicated on a scale of 1 to 5 (“1” being “Never”, “5” being “Always”) how often they wear sunscreen when they are in the sun for more than 15 minutes [25]. Self-report of sun exposure and protection behaviors have been found to be reliable and valid [34].

### 2.3. Procedures

All female psychology students were recruited during the academic school year via e-mail and web through a psychology department research subject pool at a northeastern university in the USA. Psychology 101 is a required introductory course for several majors at the university. After consenting via an online consent form, students completed the online questionnaire at their convenience. Written information about tanning and health resources was given to all participants after participation. Participants were given research participation extra credit for a psychology course and a $20 PayPal voucher as compensation for their participation.

### 2.4. Data Analysis

Data was assessed for normality, and descriptive statistics were calculated. Chi square analyses and independent samples *t*-tests were conducted first to examine differences between those who sunburned three or fewer times *versus* four or more times (based on a median split). Multivariable logistic regression analyses were then conducted to examine associations between sunburns and biological factors (fair skin, family history of skin cancer), attitudinal factors from the HBM (perceived benefits of tanning and barriers to sunscreen use, perceived severity and susceptibility to skin cancer and damage, self-efficacy for skin protection, perceived control over tanning and skin protection, and intentions to avoid tanning and protect the skin), and other behavioral factors (sunbathing and sunscreen use).

## 3. Results

Table 1 displays frequencies for each variable by number of sunburns (*i.e.*, three or fewer *versus* four or more). Compared to individuals who had sunburned three or fewer times, those who sunburned four or more times were more likely to report being fair-skinned, χ^2^(1, *N* = 509) = 50.81, *p* < 0.001, having a family history of skin cancer, χ^2^(1, *N* = 509) = 28.94, *p* < 0.001, sunbathing at least one day per week on average, χ^2^(1, *N* = 499) = 17.48, *p* < 0.001, using sunscreen more often, *t*(505) = −3.16, *p* < 0.01, perceiving greater susceptibility to skin cancer, *t*(506) = −5.92, perceiving greater benefits of tanning, *t*(494) = −6.78, *p* < 0.001, perceiving fewer barriers to using sun protection, *t*(507) = 2.87, *p* < 0.01, *p* < 0.001, perceiving lower control over tanning, *t*(506) = 5.43, *p* < 0.001, and reporting fewer intentions to avoid tanning, *t*(501) = 3.66, *p* < 0.001.

**Table 1:** Characteristics of college women by sunburn status.

Variables	Sunburned 3 or fewer times (n = 254)	Sunburned 4 or more times (n = 255)	*p*
	*n (%)*	*n (%)*	
Fair skinned	67 (26.4)	146 (57.3)	<0.001
Not fair skinned	187 (73.6)	109 (42.7)	
Family history of skin cancer	51 (20.1)	107 (42.0)	<0.001
No family history of skin cancer	203 (79.9)	148 (58.0)	
Sunbathe at least once a day/week	96 (38.6)	143 (57.2)	<0.001
Sunbathe less than once a day/week	153 (61.4)	107 (42.8)	
	*M (SD)*	*M (SD)*	
Perceived susceptibility	2.3 (1.0)	2.9 (1.1)	<0.001
Perceived severity	4.5 (0.5)	4.6 (0.5)	0.880
Perceived benefits of tanning	3.0 (1.2)	3.7 (1.0)	<0.001
Perceived barriers of sun protection	2.2 (0.7)	2.1 (0.7)	0.004
Sun protection self-efficacy	2.8 (0.9)	2.7 (0.7)	0.235
Perceived control over tanning	4.5 (2.3)	3.4 (2.3)	<0.001
Perceived control over protection	5.0 (1.8)	4.8 (1.8)	0.106
Intend to avoid tanning	5.1 (1.9)	4.5 (1.8)	<0.001
Intend to protect the skin	4.3 (1.3)	4.3 (1.3)	0.734
Frequency of sunscreen use	2.9 (1.2)	3.2 (1.1)	0.002

Multivariable logistic regression analyses showed that fairer skin (OR = 3.30, 95%CI = 2.17, 5.03), more frequent sunscreen use (OR = 1.34, 95%CI = 1.05, 1.71), greater perceived susceptibility of skin cancer (OR = 1.32, 95%CI = 1.07, 1.62), greater perceived benefits of tanning (OR = 1.39, 95%CI = 1.10, 1.76), and lower perceived control over using sun protection (OR = 0.86, 95%CI = 0.75, 0.98) were associated with being sunburned four or more times, when also taking into account family history of skin cancer, sunbathing, perceived skin cancer and damage severity, perceived barriers to using sun protection, sun protection self-efficacy, perceived control over tanning, and behavioral intentions. Results of regression analyses are reported in Table 2.

**Table 2:** Multivariable model showing associations between sunburn status and biological, attitudinal, and behavioral factors (n = 494).

Variable	OR (95%CI)	*p*
Fair skin	3.30 (2.17–5.03)	<0.001
Family history of skin cancer	1.52 (0.96–2.40)	0.073
Perceived susceptibility	1.32 (1.07–1.62)	0.009
Perceived severity	0.98 (0.67–1.44)	0.920
Perceived benefits of tanning	1.39 (1.10–1.76)	0.006
Perceived barriers of sun protection	0.70 (0.48–1.03)	0.068
Sun protection self-efficacy	0.90 (0.62–1.29)	0.552
Perceived control over tanning	0.90 (0.79–1.02)	0.101
Perceived control over protection	0.86 (0.75–0.98)	0.022
Intend to avoid tanning	1.09 (0.93–1.29)	0.295
Intend to protect skin	1.04 (0.84–1.29)	0.748
Sunbathe at least 1 day/week	1.04 (0.63–1.74)	0.872
Frequency of sunscreen use	1.34 (1.05–1.71)	0.018

## 4. Discussion

Skin cancer rates are increasing, particularly among young adult women [2,3,35]. Ultraviolet radiation, especially sunburns early in life, contribute to increased risk for skin cancer [6,7,8,9]. Despite the large literature on sun exposure and protection, the current study is one of the few to evaluate psychosocial correlates of sunburns among high risk young adult women. The data demonstrated that individuals who reported more sunburns were significantly more likely to report fair skin, higher perceived susceptibility to skin cancer and skin damage, higher perceived benefits of tanning, lower perceived control over using skin protection, and more sunscreen use. Variables not associated with greater number of sunburns were perceived severity of skin cancer and damage, sun protection self-efficacy, perceived control over tanning, intentions to avoid sunbathing or skin protection, and sunbathing. Family history of skin cancer and lower perceived barriers to skin protection were associated with a stronger history of sunburns, but these associations did not reach statistical significance in the multivariate model.

One wonders whether young adult women acquire sunburns due to biological or psychosocial factors. Based on the current data, it appears that both such factors contribute. Young women who acquire more sunburns do have fairer skin and are slightly more likely to have a family history of skin cancer but perceive greater benefits of tanning and do not engage in less sunbathing than lower risk women. Although young women who get more sunburns report lower perceived control over using skin protection, they report a higher susceptibility to skin cancer and skin damage, slightly lower perceived barriers to sun protection, and use sunscreen more often than women who get fewer sunburns. Thus, women’s sunbathing and sunscreen use behaviors may be counteracting one another in terms of sun safety. Even individuals trying to get a tan typically do not intend to burn and may be somewhat deterred from future burning [36], so the young women who are burning are likely not using sunscreen properly by applying enough broad spectrum sunscreen frequently enough, as has been shown in several prior studies [37,38,39]. In part, there may be a “teachable moment” when those who experience sunburn may initially make a greater effort to protect their skin from skin cancer and photo-aging, as was suggested by a study of students and staff at a university in Turkey [40]. However, some individuals may intend to burn in order to obtain a “base-tan,” which is not recommended.

Lower perceived control over sun protection suggests that young women who get more sunburns would like to use sunscreen more overall but do not follow through successfully when actually in a sunny environment such as a beach or pool. There seems to be a gap between education and awareness on the one hand and actual behavior on the other [41,42,43]. Future research and interventions should continue to explore this gap in order to reduce skin cancer risk among young women. Factors that may prevent behavioral follow-through could include peer pressure to sunbathe or not wear sunscreen or a lack of planning such as not purchasing or bringing sunscreen to sunny locations or reapplying after swimming [44,45]. Some studies have also used more comprehensive measures of sunscreen application that assesses amount used and re-application at appropriate intervals in order to further characterize sunscreen use behavior [25,46]. There also seems to be a conflict between the perceived benefits of tanning such as attractiveness and the desire to protect one’s health from skin damage [11]. This conflict between immediate appearance concerns and later health concerns is difficult to resolve, given that many young individuals prioritize the views of peers over authority figures and may perceive themselves as invulnerable to disease [47,48]. Further changes in societal norms regarding the attractiveness of tans and the importance of health may be required [21]. 

Strengths of the current study include the relatively large sample size and the use of the HBM. Limitations include the cross-sectional nature of the study and the inclusion of only psychology students from one US college, which may limit generalizability. However, many students are required to take psychology courses, and US college women are an appropriate study population due to their high levels of skin cancer risk behaviors and the increasing skin cancer rates among young women. An additional potential source of bias is that individuals may have been differentially motivated by the incentives provided.
